# Sequential CCR5-Tropic HIV-1 Reactivation from Distinct Cellular Reservoirs following Perturbation of Elite Control

**DOI:** 10.1371/journal.pone.0158854

**Published:** 2016-07-12

**Authors:** Sarah A. Watters, Petra Mlcochova, Frank Maldarelli, Nilu Goonetilleke, Deenan Pillay, Ravindra K. Gupta

**Affiliations:** 1 Division of Infection and Immunity, University College London, London, United Kingdom; 2 HIV Dynamics and Replication Program, NCI, Frederick, Maryland, United States of America; 3 Department of Microbiology & Immunology, University of North Carolina at Chapel Hill, Chapel Hill, North Carolina, United States of America; 4 Africa Centre for Health and Population Studies, Durban, KwaZulu-Natal, South Africa; INSERM, FRANCE

## Abstract

**Background:**

HIV Elite Controllers may reveal insights into virus persistence given they harbour small reservoir sizes, akin to HIV non-controllers treated early with combination antiretroviral therapy. Both groups of patients represent the most promising candidates for interventions aimed at sustained remission or ‘cure’. Analytic treatment interruption (ATI) in the latter group leads to stochastic rebound of virus, though it is unclear whether loss of elite control is also associated with similar rebound characteristics.

**Methods:**

We studied three discrete periods of virus rebound during myeloma related immune disruption over 2.5 years in an elite controller who previously underwent autologous stem cell transplantation (ASCT) in the absence of any antiretroviral therapy. Single genome sequencing of the V1-V4 region of env in PBMC and plasma was performed and phylogenies reconstructed. Average pairwise distance (APD) was calculated and non-parametric methods used to assess compartmentalisation. Coreceptor usage was predicted based on genotypic algorithms.

**Results:**

122 single genome sequences were obtained (median 26 sequences per rebound). The initial rebounding plasma env sequences following ASCT represented two distinct lineages, and clustered with proviral DNA sequences isolated prior to ASCT. One of the lineages was monophyletic, possibly indicating reactivation from clonally expanded cells. The second rebound occurred 470 days after spontaneous control of the first rebound and was phylogenetically distinct from the first, confirmed by compartmentalisation analysis, with a different cellular origin rather than ongoing replication. By contrast, third rebound viruses clustered with second rebound viruses, with evidence for ongoing evolution that was associated with lymphopenia and myeloma progression. Following ASCT a shift in tropism from CXCR4-tropic viruses to a CCR5-tropic population was observed to persist through to the third rebound.

**Conclusions:**

Our data highlight similarities in the viral reservoir between elite and non-controllers undergoing ATI following allogeneic transplantation. The lack of propagation of CXCR4 using viruses following transplantation warrants further study.

## Introduction

Human immunodeficiency virus (HIV) persistence in long-lived infected immune cells represents one of the major barriers to HIV eradication. It is widely thought that latently infected resting memory CD4+ T cells, established early in HIV infection, represent the primary long lived reservoir and are responsible for the rebounding virus when combined antiretroviral therapy (cART) is interrupted [1–5]. Joos et al. revealed different lineages present during analytic treatment interruption (ATI) cycles, implying stochastic reactivation of different cellular clones and therefore release of clonal virus from latently infected cells.

Elite Controllers (EC) account for 0.5–1% of infected HIV-1 individuals, and are able to control viral replication and maintain immune function for extended periods of time [6–8]. EC may reveal insights into HIV persistence given they have similar reservoir sizes compared to individuals on cART [9]. Both groups of patients represent the most promising candidates for interventions aimed at sustained remission, though there may be qualitative differences in their reservoirs. Importantly, it is not known whether loss of viral control in EC results in viral rebound characteristic is similar to that observed in ATI, for example whether rebounds are stochastic and monophyletic in nature.

We previously reported the viral kinetics and immunological correlates of viral recrudescence in an EC following myeloablation and autologous stem cell transplantation (ASCT) in the absence of ART [10] and (S1 Fig). Following this initial viral rebound, immune mediated control was achieved with kinetics similar to that of three drug ART and maintained for more than a year. The individual subsequently presented with relapsed multiple myeloma and was found to have loss of HIV-1 control. Here we have analysed in detail the rebounding virus between the initial ASCT and ensuing periods of relapsed myeloma.

## Materials and Methods

### Nucleic acid extraction and single genome sequencing

We used single genome sequencing (SGS) of the V1-V4 region of env as described [11, 12]. This method is unbiased by PCR introduced recombination or resampling of sequences [11, 13, 14].

Viral RNA was extracted from plasma using the QIAamp Viral RNA Kit (Qiagen, Hilden, Germany) and reverse transcribed at 50°C for 60 min followed by 55°C for 60 min using (10 units/ul) SuperScript III (Invitrogen, California, USA) in the supplied 1× RT buffer with 0.5mM dNTPs, 5mM DTT, 2 units/**μ**l RNaseOut (Invitrogen, California, USA) and 0.25**μ**M of OMF19 [11]. Following reverse transcription the SuperScript III was inactivated at 85°C for 10 min, and the cDNA was treated with 2 units of RNaseH (Invitrogen, California, USA) at 37°C for 30 min.

Viral DNA was extracted from peripheral blood mononuclear cells (PBMCs) which were isolated from 50ml EDTA blood using a standard Ficoll-hypaque separation method utilizing Lymphoprep (STEMCELL Technologies, Cambridge UK). Total DNA was then extracted from PBMCs using the Qiagen DNeasy kit (Qiagen, Hilden, Germany).

PCR reactions were carried out using High Fidelity Platinum *Taq* DNA polymerase (Invitrogen, California, USA) and primers Env5out and OMF19 [11], followed by primers Env5in and Env3in for the nested PCR. The PCR amplifications were carried out in 1× High Fidelity Platinum PCR buffer, 2mM MgSO_4_, 0.2mM dNTPs, 0.2**μ**M of each primer and 0.025 units/**μ**l Platinum *Taq*. The PCR conditions were as follows: 94°C for 2 minutes, followed by 35 cycles of 94°C for 15 seconds, 55°C for 30 seconds and 68°C for 4 minutes, finishing with a 10 minute elongation step at 68°C. All PCR products were run on precast 1% agarose E-gel 96 (Invitrogen, California, USA) and then the V1-V4 region of the HIV envelope was sequenced using primers REV16 and FOR14 [12].

### Sequence analysis

Chromatograms were manually checked for the presence of mixed bases, any sequences containing mixed bases, indicating the presence of multiple templates, were excluded from further analysis. All sequences were submitted to Genbank and can be found under accession numbers KX267967—KX268088. Sequences were assembled using an in-house program and aligned in MEGA 5.2 (http://www.megasoftware.net) [15]. Neighbour joining (NJ) trees were also made in MEGA 5.2 and branch support was tested by 1000 bootstraps. Maximum Likelihood trees were made using the GTRGAMMA model in RAxML [16] branch support was inferred by 1000 bootstraps. All trees were rooted on an outgroup, the molecular clone MJ4 [17]; in order to rule out contamination a separate NJ tree was made that included commonly used plasmids within our laboratory (data not shown). Trees were annotated and edited using FigTree [18]. Population genetic diversity and divergence were calculated as average pairwise difference (APD) in MEGA 5.2. The presence of hypermutants was determined using Hypermut software within the Los Alamos website [19].

We assessed population shifts (geographic subdivision), and migration using the non-parametric tree based methods; Simmonds Association Index (SAI) [20], Slatkin Maddison (SM) [21]. In addition we used two nucleotide distance based methods, which do not rely upon phylogeny, Wrights FSt and Nearest Neighbour (Snn). Distances were estimated using a maximum likelihood approach under a general time reversible (GTR) nucleotide substitution model, estimating all parameters independently for each branch. The four tests were performed within HyPhy [22]. To predict viral tropism we used two genotypic algorithms Geno2pheno [23] and Phenoseq [24, 25]. Concurrence between both programs was required in order to assign CXCR4 tropism to a particular *env* sequence.

### Quantification of HIV nucleic acids

HIV-1 RNA and total HIV-1 DNA were quantified as previously described [10, 26].

### Ethics statement

Ethical approval for this research was granted by the NRES (National Research Ethics Service) Committee London, Harrow, UK (Research Ethics Committee (REC) number 12/LO/0405). Written, informed consent was obtained from the study participant and recorded on a paper consent form that was approved by the NRES Committee London, Harrow, UK.

## Results

### Perturbations in elite control correlate with myeloma relapses

**Fig 1:**
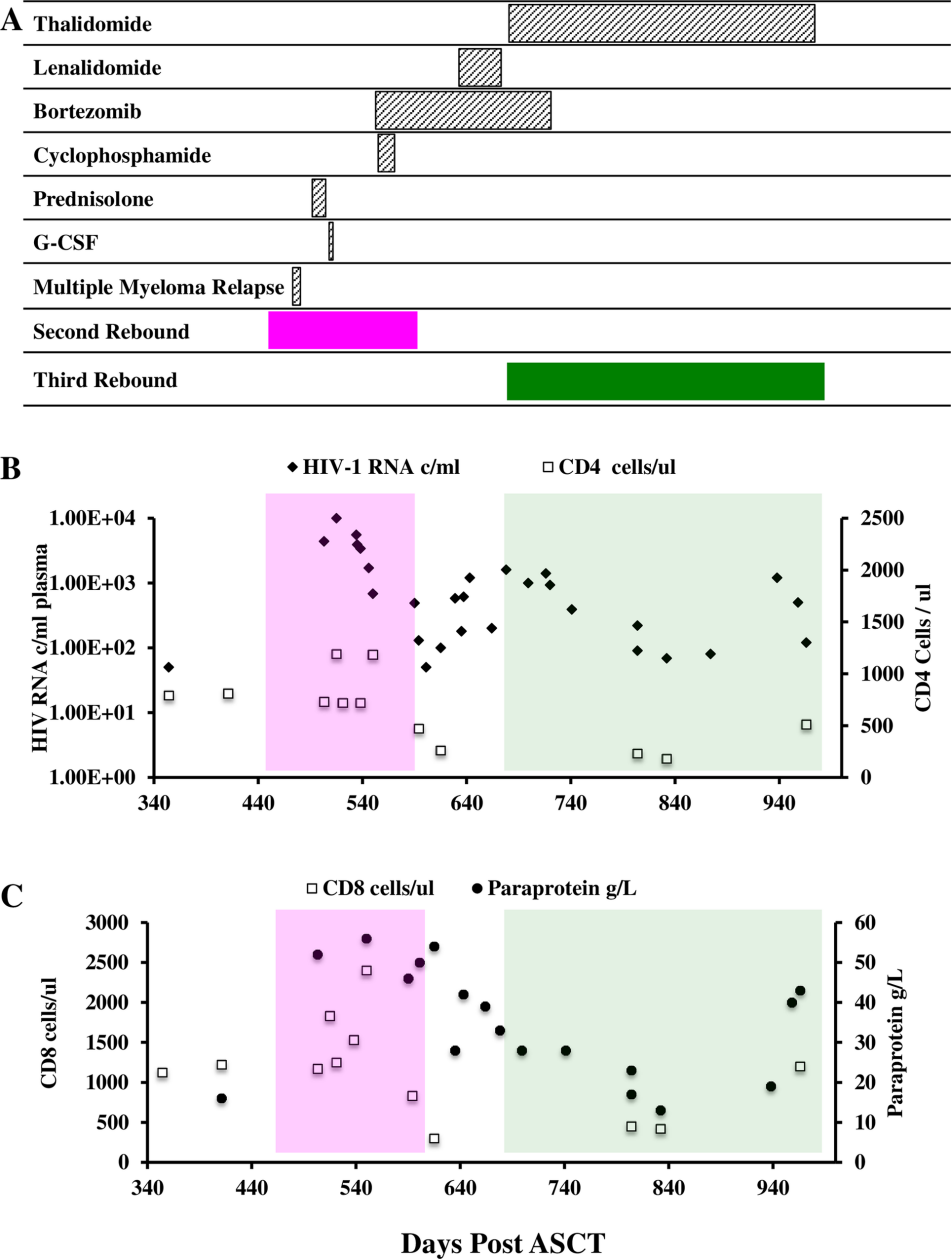
**HIV viral rebound following relapsed myeloma** (A) Diagram representing prescribed drugs known to have immunological effects extended horizontally to represent time and correspond with days post ASCT as represented in graphs (B) and (C). Additionally the time of myeloma relapse and the second (magenta) and third (green) rebound periods are highlighted. (B). Graphical representation of longitudinal HIV plasma RNA viral load and CD4+ T-cells counts. Magenta and green shaded boxes indicate the second and third rebound time points and correspond to (A). (C). (A) scatter plot of longitudinal CD8+ T cell count and paraprotein levels over days post ASCT, the shaded boxes correspond to (A).

As previously reported [10], the study subject experienced viral recrudescence, peaking at 28,000 c/ml, with spontaneous resolution by day +37 following ASCT (S1 Fig). Fig 1 illustrates HIV viral load (c/ml), CD4+ and CD8+ counts (cells/ul), as well as paraprotein levels (g/L) during the period of days +354 to +966 post ASCT. On day +503 routine tests revealed a HIV-1 plasma viral load of 4400 c/ml, HIV-1 total DNA of 100 c/1e^6^ PBMCs, a CD4+ count of 730mm^3^ (25%) and CD8+ cell count of 1170mm^3^ (40%) whilst paraprotein was noted to have increased to 52g/L. At this point a diagnosis of biochemical relapse of multiple myeloma was made. The viral load steadily increased and at day +521 peaked at 12,000 c/ml in plasma, with a doubling time of 10.5 days (in contrast to 0.5 days for the first rebound). The individual was given two cycles of bortezomib (2.75mg), dexamethasone (20mg) and cyclophosphamide (500mg), followed by four cycles of bi-weekly bortezomib (2.75mg) (Fig 1A). The paraprotein levels did not improve in the short term and HIV-1 viral load declined over the following weeks reaching <50 c/ml by day +601 (Fig 1B and 1C). The decay rate was much slower than that observed following the initial rebound associated with ASCT. Further treatment for myeloma with lenalidomide was commenced and later substituted for thalidomide (100mg) on day +635 due to an adverse drug reaction. Over the following months the viral load followed a fluctuating course, mirroring paraprotein levels (Fig 1B). Despite paraprotein levels reaching 13g/L and HIV viral load declining to 69 c/ml on day +832, paraprotein levels and HIV viral load went on to steadily increase. During this disease course the individual was offered and refused cART. Unfortunately the individual succumbed to multiple myeloma on day +1004.

### Single genome amplification reveals stochastic HIV-1 rebound from limited sources

**Fig 2:**
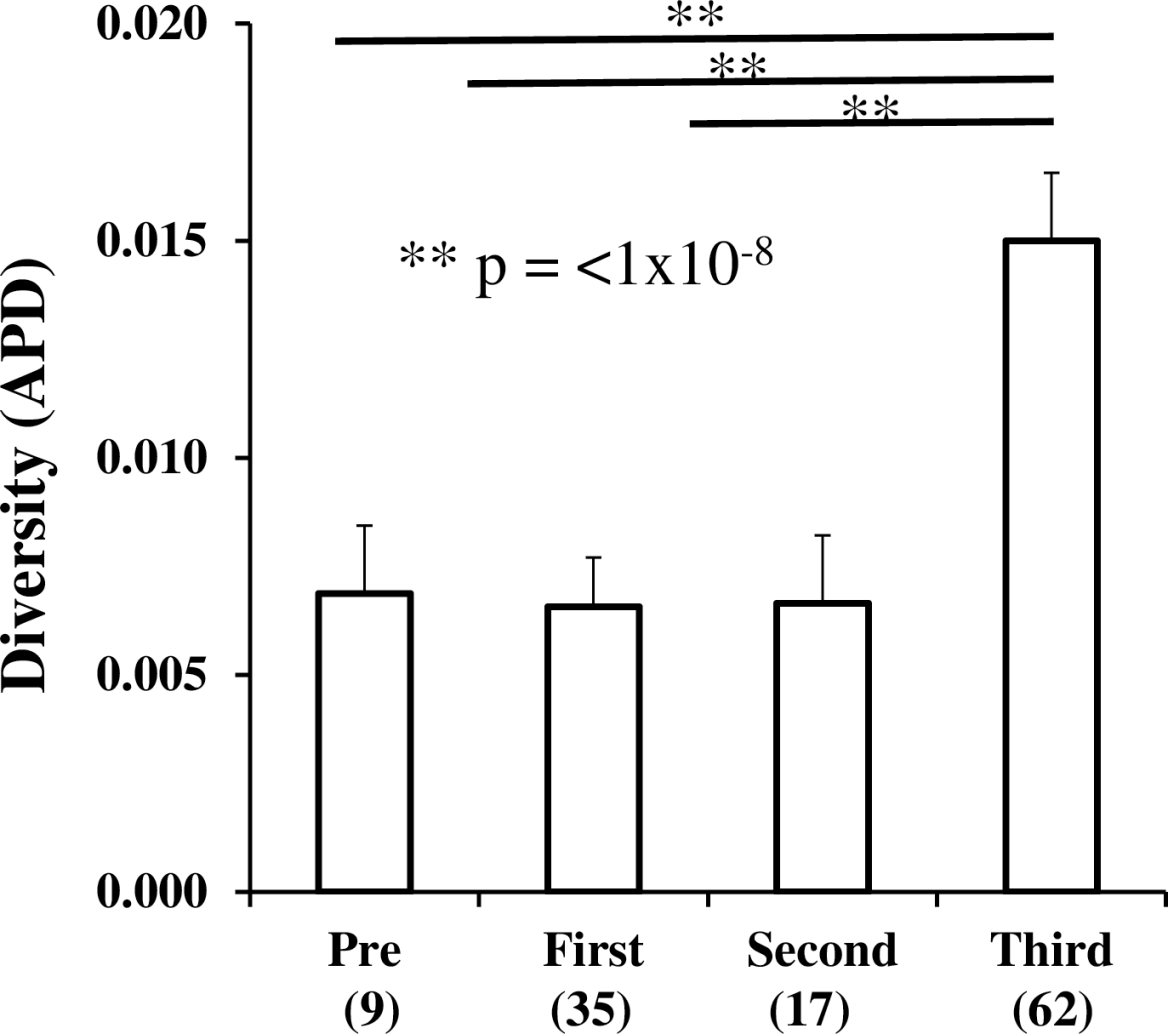
Diversity measured as average pairwise distance at longitudinal time points. APD measured pre- ASCT and during subsequent viral rebounds. Numbers in parentheses indicate the number of single genomes acquired at each time point. Significance was determined by two-tailed.

**Fig 3:**
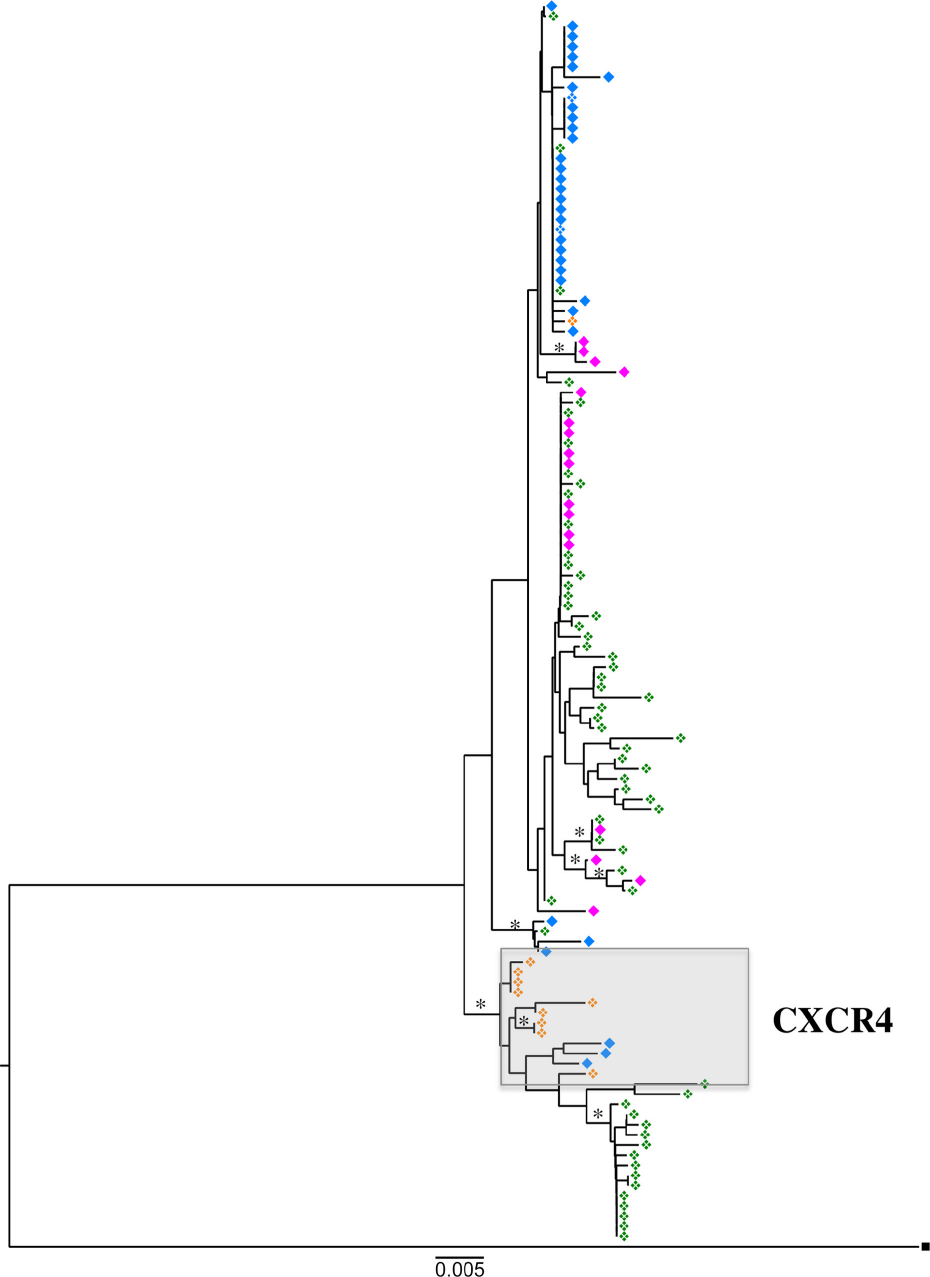
Inferred neighbour-joining phylogenetic tree of single genome derived HIV-1 env sequences. Two APOBEC hypermutated sequences found in the third rebound have been removed. Pre melphalan HIV DNA (-27 to -38, orange open diamonds), first rebound HIV RNA (+6, blue closed diamonds), first rebound HIV DNA (+6, blue open diamonds), second rebound HIV RNA (+515, magenta closed diamonds), third rebound HIV DNA (+643 to +958, green open diamonds). Rooted on MJ4 (black square), branches with bootstrap support > 75% are indicated by black asterisk. Overall population APD is 1.5%. The scale represents 0.005 nucleotide substitutions per site, equivalent to 4.5nt. The tips in the boxed grey area representative sequences predicted to confer CXCR4 tropism by both Geno2Pheno and Phenoseq genotypic algorithms.

We obtained a total of 122 single genome *env* sequences, with a median of 26 sequences per rebound (range 10–62, Fig 2). Average pairwise distance (APD) did not increase between first and second rebound time points relative to pre-ASCT (Fig 2). Phylogenetic analysis, depicted in Fig 3 and S2 Fig, of *env* sequences isolated prior to ASCT (open orange diamonds) indicated that rebounding plasma *env* sequences (blue diamonds) represented three distinct clusters and this compartmentalization of rebound was confirmed by four non-parametric analyses, shown in Table 1. One of these plasma RNA derived clusters consisted of a large monophyletic population, possibly, consistent with viral rebound from a single latently infected cellular clone (Fig 3, blue diamonds upper part of tree). These data demonstrate virus and cellular persistence despite treatment with melphalan and ASCT.

**Table 1:** Non-parametric geographic subdivision tests comparing pre- ASCT viruses with those at each rebound.

	SM		SAI		Wrights FSt	Nearest Neighbour (Snn)
Time Point	ME (<11)	*p* (<0.05)	AI (<0.6)	BS (>95)	*p* (<0.05)	*p* (<*0*.05)
**Pre–ASCT vs First Rebound**	3	0.000	0.131	100	0.000	0.001
**Pre–ASCT vs Second Rebound**	2	0.000	0.124	100	0.000	0.001
**Pre–ASCT vs Third Rebound**	6	0.000	0.176	100	0.134	0.000
**First Rebound vs Second Rebound**	4	0.007	0.554	97	0.142	0.001
**First Rebound vs Third Rebound**	6	0.000	0.231	100	0.257	0.000
**Second Rebound vs Third Rebound**	8	0.403	0.68	89	0.689	0.084

SM- Slatkin Maddison, SAI–Simmonds Association Index, ME: Migration Events, AI: Association Index, BS: Bootstrap Significance (1000). Wrights FSt. *p*: Probability based on 10,000 permutations was computed, with the permutation test randomly allocating sequences into each group compared. Hypermutants removed and identical sequences collapsed.

In order to determine if the rebounding viruses isolated during relapse of multiple myeloma (second and third rebounds, pink and green shaded area Fig 1A) had diverged from those isolated during the initial rebound, we next analysed *env* sequences from five time points, the first being prior to the second peak viraemia on day +515 and the last at day +958 (Fig 3).

*Env* sequences obtained from plasma RNA during the second rebound (day +515, Fig 3, depicted by pink symbols) form two separate clusters from the majority of *env* sequences obtained from plasma RNA during the first rebound (day +6). The phylogenetic tree indicated divergence between the viral populations sequenced from the first and second rebounds, possibly indicating plasma viruses originating from separate cellular populations (Fig 3 and S2 Fig). This change in the viral population was confirmed by compartmentalisation analysis (Table 1).

The majority of RNA sequences obtained from the second rebound were found together as a monophyletic rake of eight identical sequences, accounting for 47% of the second rebound *env* sequences sampled. Possibly suggesting that these RNA sequences had the same cellular origin.

The genetic distance, measured as APD, increased from 0.7% to 1.5% (Fig 2) during the third rebound period, indicating more widespread replication from multiple sources. This was co-incident with myeloma progression, possibly indicating further immune compromise and loss of viral control.

Corresponding with the marked increase in APD during the third rebound, proviral *env* sequences obtained from PBMC during the same period (Fig 3 –open green diamonds) intermingled with RNA sequences obtained from plasma during the second rebound, supporting viral replication at this later time point, rather than activation of latently infected cells during this period.

Importantly, there were two clusters of identical sequences from the second and third rebounds, consistent with persistence of cellular clones during chemotherapy. As predicted, compartmentalisation analysis of sequences obtained during the second and third rebound (Table 1) did not demonstrate evidence of population shift or migration during this time.

### Shift in coreceptor usage from CXCR4 to CCR5 following myeloablation

**Fig 4:**
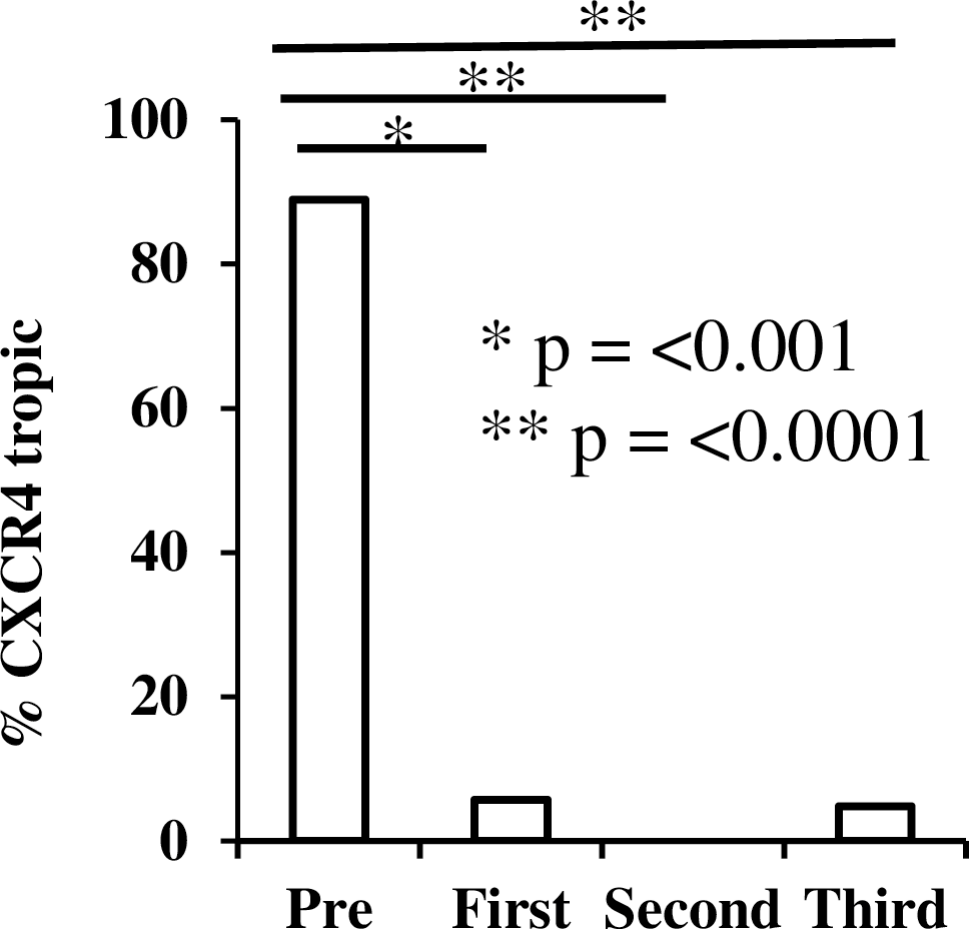
Genotypically defined co-receptor usage following HIV rebounds. Tropism testing of the HIV V3 loop was performed in both Geno2Pheno and Phenoseq online genotypic algorithms. Percentage of CXCR4 sequences is plotted on the Y-axis. Only sequences found to be X4 in both programs were designated as X4. Significance was determined using a two-tailed Fishers Exact test.

88.9% of proviral sequences, obtained an average of 36 days (range 27–44 days), prior to the ASCT demonstrated genotypic tropism for the co-receptor CXCR4 (Figs 3 and 4). By contrast, during the initial rebound following ASCT only 5.7% of the RNA virus found in plasma demonstrated genotypic tropism for CXCR4 (Fig 3 and Fig 4), suggesting that CCR5-using virus preferentially reactivated and replicated or that cells harbouring CCR5 virus preferentially survived the transplantation process. Sequences obtained from the second rebound were 100% CCR5 tropic and the third rebound was also dominated by CCR5 using viruses (95.2% CCR5), even in the context of a depressed CD4 count (180 cells/uL).

## Discussion

We observed stochastic reactivation of virus replication in an individual with natural control of HIV-1 during immune disruption occurring over a period of 2.5 years. Compartmentalised, monophyletic HIV-1 RNA *envs* from the first and second rebounds suggest similarities in the viral reservoir between EC and non-controllers undergoing treatment interruption following allogeneic transplantation, as reported by Henrich and colleagues [27]. In the aforementioned study, the authors deduced that only one, or a small number of cells/latent proviruses, contributed to viral rebound after ATI.

Viral diversity in our individual was low and remained stable until the third rebound of plasma virus, when diversity increased from 0.7% to 1.5%. This may reflect a global loss of control that was likely multi-factorial. During the third rebound the individual was treated with thalidomide for relapse of multiple myeloma and his CD4+ T cell count was at its lowest (around 200 cells /ul). Additionally, and importantly, during the third rebound the viral load did not become fully suppressed, the lowest viral load being 69 c/ml (Fig 1). Therefore, the third rebound likely represents the result of ongoing viral replication rather than activation of latently infected cells. This is supported by the phylogeny in which we observe third rebound viruses to be well mingled between other rebound time points, and further supported by geographic subdivision analysis which did not identify the third rebound as emerging from an individual compartment. Immunomodulatory agents (IMiDs) have been reported to activate T cells [28–30], this individual received lenalidomide and thalidomide prior and during the third rebound which may partly explain our observation of increasing viral diversity.

Finally, we observed an unexpected parallel between our case and the ‘Berlin patient’, the only individual thought to have achieved HIV ‘cure’. Tropism testing of the viral population within the Berlin patient [31] prior to transplant with a homologous CCR5 delta32 donor revealed a minority (2.9%) of X4 tropic species within plasma RNA, but not in proviral DNA. This individual has not experienced virus rebound despite the presence of X4 virus in plasma. In our individual, genotypic tropism testing of proviral sequences obtained pre-transplant revealed a majority X4 population. Following ASCT a shift in tropism from CXCR4 using proviruses to a population mostly using CCR5 occurred, despite a minority X4 species remaining in RNA as well as DNA, indicating that these viruses were replication competent. It could be that the X4 viruses were not as fit as the R5 viruses that rebounded in our individual, or that there was a larger reservoir of R5 pre-transplant proviruses in an unsampled compartment such as lymph node. Another possibility is that cells harboring X4 viruses are less likely to survive the effects of chemotherapy. Finally one should consider the possibility that the rebound originated in myeloid cells infected with R5 tropic virus.

## Conclusion

Although the data are derived from a single case, they highlight similarities in the viral reservoir between elite and non-controllers undergoing ATI following stem cell transplantation. Insights from rare individuals such as EC will continue to be very informative in elucidating the nature of HIV-1 reservoirs as we search for a curative HIV intervention. Given the preferential reactivation of CCR5 tropic viruses in both our case and previous ATI studies, CCR5 antagonists may be useful in curative interventions even where CXCR4 and CCR5 variants coexist.

## Supporting Information

S1 Fig Dynamic changes in HIV nucleic acids and total lymphocytes following ASCT. Administration of melphalan at day 0. (A) Longitudinal PCR quantification of HIV RNA and DNA, and total lymphocyte numbers following the first 50 days post ASCT. (B) Plasma HIV-1 RNA over time ASCT. First and second phase decay is displayed by dotted lines.
(TIF)

S2 Fig Inferred maximum likelihood phylogenetic tree of single genome derived HIV-1 env sequences. Identical sequences have been collapsed and a single representative sequence left in the alignment to build the tree. Identical sequences have then been extended horizontally across the tree at the tip at which they appeared. Pre melphalan HIV DNA (-27 to -38, orange open diamonds), first rebound HIV RNA (+6, blue closed diamonds), first rebound HIV DNA (+6, blue open diamonds), second rebound HIV RNA (+515, magenta closed diamonds) and third rebound HIV DNA (+643 to +958, green open diamonds). Rooted on MJ4, indicated by a black square. Branches with bootstrap support > 75% are represented by black asterisk. Open squares represent APOBEC hypermutated sequence. The overall population has a APD of 1.5% (with the hypermutants removed). The scale represents 0.005 nucleotide substitutions per site, equivalent to 4.5nt.
(TIF)
