# Supplementary figures and images for: Sequential CCR5-Tropic HIV-1 Reactivation from Distinct Cellular Reservoirs following Perturbation of Elite Control
Source: PLoS One. 2016 Jul 12;11(7):e0158854. doi: 10.1371/journal.pone.0158854 (PMC4942039; doi:10.1371/journal.pone.0158854)

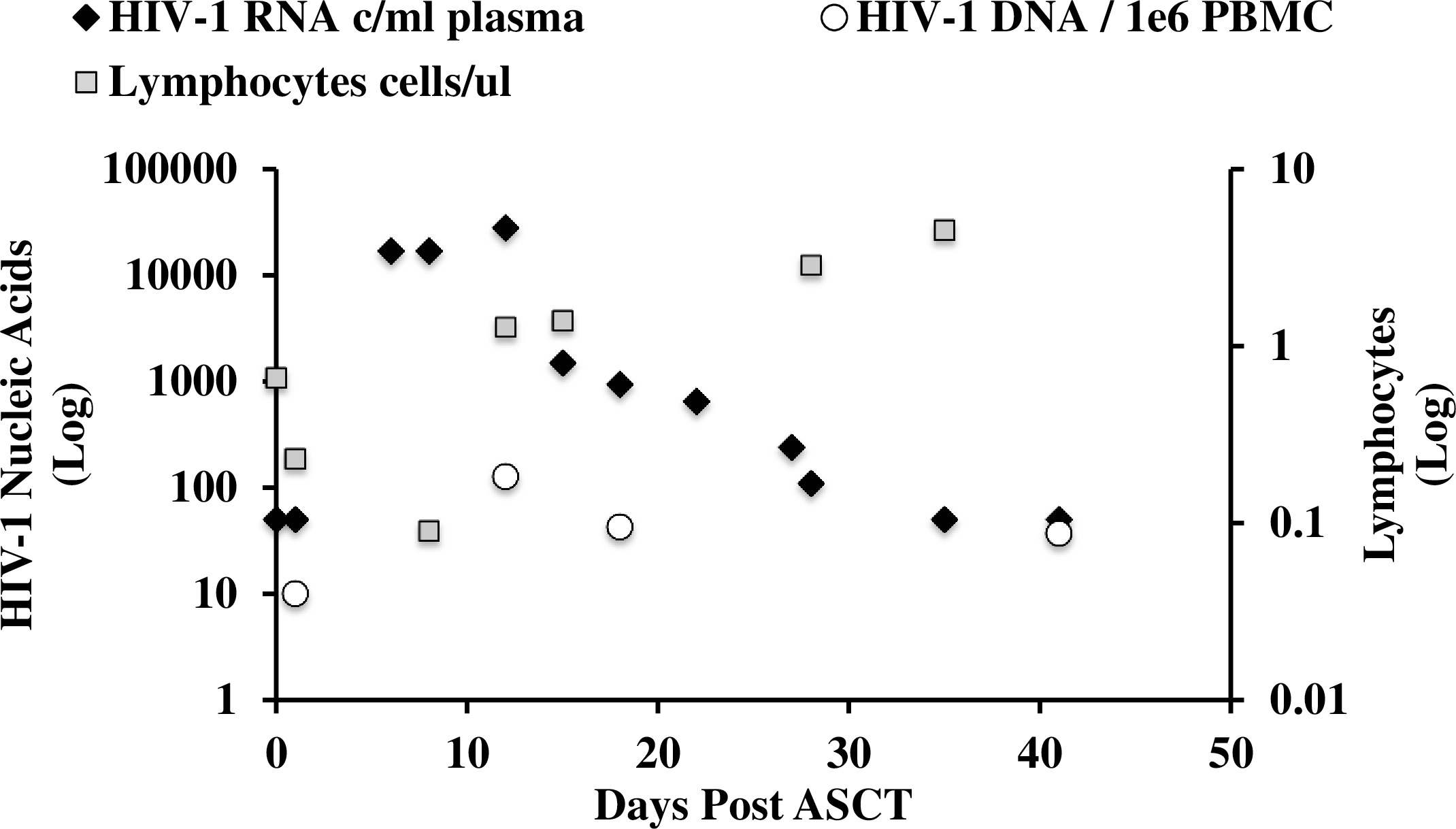

Supplement: S1 Fig — Administration of melphalan at day 0. (A) Longitudinal PCR quantification of HIV RNA and DNA, and total lymphocyte numbers following the first 50 days post ASCT. (B) Plasma HIV-1 RNA over time ASCT. First and second phase decay is displayed by dotted lines. (TIF) [file pone.0158854.s001.tif]

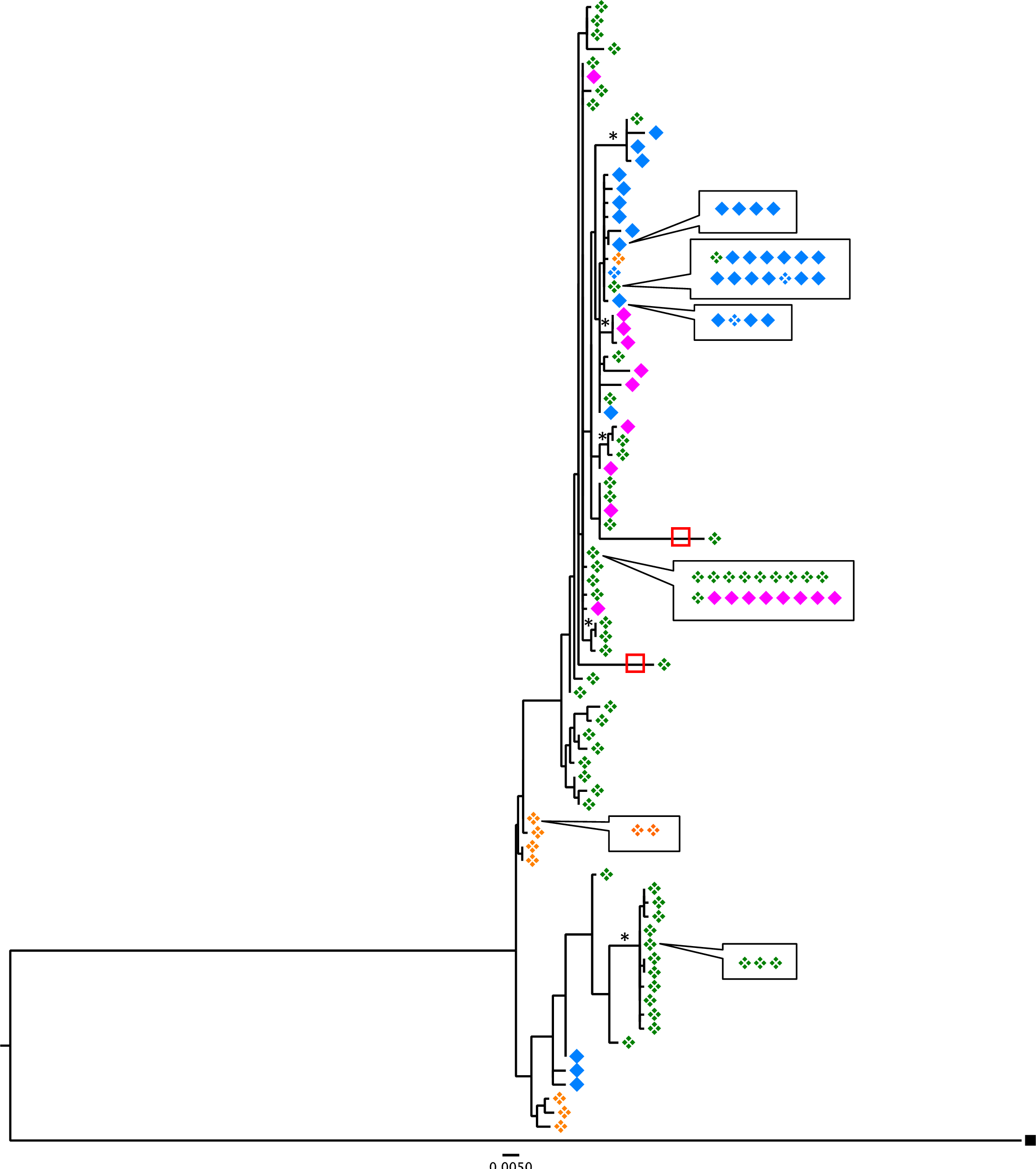

Supplement: S2 Fig — Identical sequences have been collapsed and a single representative sequence left in the alignment to build the tree. Identical sequences have then been extended horizontally across the tree at the tip at which they appeared. Pre melphalan HIV DNA (-27 to -38, orange open diamonds), first rebound HIV RNA (+6, blue closed diamonds), first rebound HIV DNA (+6, blue open diamonds), second rebound HIV RNA (+515, magenta closed diamonds) and third rebound HIV DNA (+643 to +958, green open diamonds). Rooted on MJ4, indicated by a black square. Branches with bootstrap support > 75% are represented by black asterisk. Open squares represent APOBEC hypermutated sequence. The overall population has a APD of 1.5% (with the hypermutants removed). The scale represents 0.005 nucleotide substitutions per site, equivalent to 4.5nt. (TIF) [file pone.0158854.s002.tif]
